# Photobiomodulation Therapy: The Dawn of Myopia Control

**DOI:** 10.3390/cells15060526

**Published:** 2026-03-16

**Authors:** Kate Gettinger, Yinuo Huang, Kazuo Tsubota, Kazuno Negishi, Toshihide Kurihara

**Affiliations:** 1 Laboratory of Photobiology, Keio University School of Medicine, 35 Shinanomachi, Shinjuku-ku, Tokyo 160-8582, Japan; 2 Department of Ophthalmology, Keio University School of Medicine, 35 Shinanomachi, Shinjuku-ku, Tokyo 160-8582, Japan; 3 Tsubota Laboratory, Inc., Keio Hospital, 35 Shinanomachi, Shinjuku-ku, Tokyo 160-8582, Japan

**Keywords:** myopia, photobiomodulation, myopia prevention, nonvisual opsins

## Abstract

**Highlights:**

**What are the main findings?**

- Light exposure potentially influences myopia development through a variety of proposed mechanisms, although a full understanding of the mechanisms is still lacking.
- Recent research into the role of nonvisual opsins and spectrum-specific influences on myopia development has highlighted the complexity of these potential pathways.

**What are the implications of the main findings?**

- Future myopia prevention strategies may utilize promising findings featuring both red, blue, and violet light exposure as a means to control axial growth and refractive changes.
- While there is still much to be explored, recent findings have suggested several potential mechanisms through which light influences myopia development.

**Abstract:**

As the prevalence of myopia, or near-sightedness, continues to rise globally, it becomes imperative to determine the mechanisms driving myopia so that appropriate interventions to mitigate it can be developed. Light appears to be critical for normal ocular development, and over the past several decades research has explored the connection between light exposure and myopia development. This review explores the growing field of photobiomodulation, or the use of light to modulate biological processes, to prevent myopia development. To complete this review, relevant texts published from January 1990 to December 2025 were retrieved from the PubMed database using a combination of search terms covering myopia and ocular development, light exposure conditions related to myopia, myopia development in relation to circadian and diurnal regulation, nonvisual opsins and myopia, and light-induced ocular damage. Through this review, we see that photobiomodulation offers a potential intervention to control myopia progression, but the mechanisms behind light’s influence on ocular development remain complex and incompletely understood. This review aims to summarize what is currently known to serve as a basis for future research and to delineate important findings.

## 1. Introduction

Light has been shown in numerous studies to have a significant impact on our well-being [1], as well as our cognitive function [2]. Research is continually expanding to explore its applications to human health, including its role in both the treatment and prevention of diseases. Over the past several decades, numerous studies have successfully correlated light exposure with the development of myopia, although the mechanisms driving this relationship are still not fully elucidated.

Myopia, or nearsightedness, is predicted to affect approximately one-third of children and adolescents globally by 2050 [3]. A higher proportion of myopia is seen in East Asia and urban areas, as well as among women, adolescents, and high school students [3]. In Japanese junior high school students, myopia prevalence is as high as 95%, while high myopia has a prevalence of 11% [4]. Meanwhile, in adults over 40 years old, the prevalence of high myopia is estimated at around 5%, further indicating that in Japan, especially, the prevalence of severe myopia is rapidly rising [5]. By the year 2050, over 740 million people are predicted to have myopia [3]. Global estimates of vision impairment and blindness identify uncorrected refractive error as a leading cause, underscoring the broader public health relevance of reducing myopia progression [6].

Compared to emmetropes, those with high myopia have a significantly increased risk of visual impairment over their lifetime [7], with vision loss typically due to conditions such as myopic macular degeneration [8] and glaucomatous and non-glaucomatous optic neuropathy [8,9]. High myopes are also at greater risk of retinal detachment, chorioretinal atrophy, and optic disc abnormalities [10]. Beyond these physical ocular complications, myopia also imposes a psychological burden, particularly in adolescents. A study of first-year high school students in Guangzhou found a significant correlation between myopia, especially more severe myopia, and increased anxiety levels, highlighting its impact on mental health [11].

Despite its known complications and widespread prevalence, a comprehensive understanding of the mechanisms driving myopia, as well as strategies to mitigate it, are still lacking. Light exposure has been noted as a potential cue for modulating refractive development. Initial insights from epidemiological studies showed that children who subjectively spent more time outdoors had lower myopia prevalence, even when accounting for parental levels of myopia [12] or excessive amounts of near work [13]. While several subsequent studies further supported a connection between increased outdoor time and reduced myopia incidence [14,15,16], meta-analyses of such studies indicated notable variability in the amount of change in refractive error and axial length [17,18]. Some studies also found that the influence of outdoor time appeared to be less effective in children who were already myopic [14,19,20]. While these epidemiological studies had limitations, for instance, many relied on subjective reporting of outdoor time and occasional incidences of publication bias were exhibited, in addition to difficulty in ensuring adherence in large study populations, the collective evidence still showed an encouraging trend of increased outdoor time being correlated with slowed myopia onset. However, it was still unclear what aspect of outdoor time may be responsible for reducing myopia incidence. One of the early hypotheses was that the higher-intensity light experienced outdoors may modulate ocular growth by stimulating dopamine release, leading to slowed axial growth [13]. Animal studies seemed to support this idea by providing evidence of high-intensity light slowing and blocking experimental myopia [21,22,23,24]. Since then, a number of other explanations have been offered for why increased time outdoors may be linked to reduced myopia. These include differences in spatial frequency between indoor and outdoor settings [25], reduced exposure to peripheral defocus in outdoor settings [26], less near work with outdoor activity [27], varying chromatic defocus signals [28], and the varying spectral composition of indoor versus outdoor light [24]. While some of these ideas have received more traction than others, systematic reviews have generally indicated that most studies support the role of light influencing eye growth and refractive development in some way [29,30]. However, it remains unclear whether it is only particular aspects of light, such as intensity or spectral composition, which influence refractive development, and the mechanisms through which it operates are still largely unknown. While interest in light-based therapies for treating and preventing myopia is rising, it remains difficult to establish any evidence-based clinical guidelines. This is in part due to a lack of randomized controlled trials in humans, but also due to the inherent challenges in translating animal studies to humans. Although there has been substantial progress in the field of photobiomodulation, or the use of light to influence biological processes, there are still notable knowledge gaps that need to be addressed before it can be used to influence myopia. The aim of this review is to summarize and evaluate current peer-reviewed published evidence supporting light exposure as a way to influence refractive development and ocular growth. In the process, it also attempts to identify current gaps in knowledge and limitations in the current research field in the hope of helping to guide future research.

This comprehensive review was conducted by searching peer-reviewed published evidence and applicable authoritative texts published from January 1970 to February 2026. The keywords “photobiomodulation”, “nonvisual opsins”, “myopia prevention”, “myopia”, and “light and myopia” were used, as well as a combination of terms that covered myopia and ocular development, light exposure conditions relating to myopia, circadian and diurnal regulation in relation to myopia development, and light-induced ocular damage. Additional relevant studies were identified by reviewing references cited in, or citing, the initially included studies. The inclusion criteria consisted of studies published within peer-reviewed journals, including animal and human studies in children to young adults. Any non-English language studies were excluded. The aim of this review was to integrate outdoor light exposure, spectral properties, circadian rhythm, and interventional studies to compile the latest understanding of photobiomodulation.

## 2. Light and Myopia: A General Overview

Early animal studies provided evidence that light appears to be critical for normal ocular development. In a lens-induced myopia (LIM) model in chicks, chicks showed a dose–response relationship between the amount of reduced myopia induction and the duration of exposure to high-intensity light, with 6 h having the strongest effect on preventing myopic shifts and axial elongation [31]. Further chick studies demonstrated that the level of protection from form-deprivation myopia (FDM), another common animal myopia model, correlates with exposure to increasing light intensities [32]. In tree shrews undergoing FDM and LIM [33] and chicks with FDM [21] or LIM [23], increased laboratory lighting slowed myopia development. Guinea pigs have also shown similar correlations between bright light (10,000 lux) and reduced FDM-induced myopia [34], as have rhesus monkeys reared in 18,000 to 28,000 lux bright light [24]. Sufficient light–dark cycles also appear to be critical, with at least 4 h of consistent darkness necessary for emmetropization to occur in chicks, hinting at the potential for circadian involvement [35]. These early findings implicated light’s role in refractive development and encouraged further studies.

The results of animal studies like these were corroborated by observational evidence in humans. However, due to the experimental limitations of human studies, most findings from human studies merely provide an association between light and refractive development and are unable to provide the convincing correlations permitted by animal studies. At the behavioral level, longitudinal cohort data has indicated that children who develop incident myopia spend less time outdoors than those who remain non-myopic, supporting daylight exposure as a plausible protective correlate [36]. However, it could be argued that simply being myopic or wearing a myopic prescription may influence a child’s behavior, such as preferring indoor near-vision tasks as opposed to outdoor play. Further investigations attempted to draw more direct correlations between outdoor light exposure and myopia. In a prospective, 18-month study of 101 children aged 10–15 years, personal illuminance was recorded using wrist-worn light sensors during two separate 14-day monitoring periods and related to axial length changes across four visits; higher average daily light exposure was associated with slower axial elongation, even after adjustment for relevant covariates [37]. Among school children, it was demonstrated that simply spending more time outdoors was associated with a reduced risk of myopia in young adulthood [17]. In another study, Indian schoolchildren between the ages of 9 and 15 wore tracking sensors to record light exposure continuously over six days and quantify illuminance, outdoor time, and outdoor “epochs” (the number of times a participant was exposed to ≥1000 lux thresholds) for comparison [38]. While overall differences were modest, non-myopes showed significantly higher illuminance exposure on non-school days, and during travel to and from school non-myopes had higher illuminance exposure, spent more time outdoors, and had more outdoor epochs than myopes, suggesting small but influential exposure differences [38]. This inverse relationship between myopia and light exposure is also observed in young adults. Conjunctival ultraviolet autofluorescence, an objective marker of ocular sun exposure, was inversely associated with myopic refractive error, with the lowest exposure quartile having more than double the prevalence of myopia compared to the highest quartile [39].

Furthermore, seasonal and photoperiod studies provided similar evidence consistent with a role for natural daylight; myopia progression and axial elongation progress slower in summer than in winter in cohorts of school-aged children [40,41]. In Danish myopic children, greater accumulated daylight hours were correlated with less axial growth and less myopia progression, while shorter-day periods showed greater elongation and refractive change [42].

In addition to natural light exposure, indoor lighting conditions may also be relevant. In the Beijing Childhood Eye Study, dim reading illumination was associated with myopia outcomes in multivariable analyses [43]. A compelling natural experiment emerged during the coronavirus disease 2019 (COVID-19) pandemic. The COVID-19 home quarantine created a global situation in which children’s outdoor time decreased and myopia progression accelerated. Although screen exposure time showed a stronger statistical association with myopia development, the overall low outdoor time in this cohort, together with the possibility that increased screen use altered light exposure, still implies a potential protective role of daylight-related behaviors [44].

Building upon these association findings, intervention studies have aimed to test whether increasing light exposure can prevent myopia. In a randomized clinical trial in China, adding 40 min of outdoor activity at school reduced the incidence of myopia over a 3-year period [45]. Similarly, after imposing a new policy encouraging schools to take students outside for 120 min per day, Taiwanese schools saw a reversed trend in reduced vision [46]. However, this study did not measure refraction, so it cannot be directly concluded that the outdoor time specifically reduced myopia. Meanwhile, a cluster-randomized intervention controlled trial in Taiwan among grade 1 schoolchildren implemented a school-based outdoor promotion program with objective light-meter recordings, with the result being significantly less myopic shift and axial elongation over one year in the intervention group compared with controls [47]. Importantly, analyses incorporating illuminance thresholds (e.g., ≥1000 lux and ≥3000 lux) suggested that strong sunlight exposure may not be necessary and that increased outdoor time in relatively lower-intensity settings, such as under trees or in hallways, may also reduce myopia progression [47].

The biological effect of outdoor time is rapid, as evidenced by short-term studies. A Japanese study demonstrated that there are even short-term ocular changes associated with outdoor time [48]. Among school children spending 6 h outdoors participating in “intense” outdoor activities, choroidal thickness increased after just one week of the intervention [48]. These cumulative findings make it evident that spending more time outdoors has a positive influence on refractive error, while less light exposure leads to more myopic outcomes.

However, it is worth noting that there is a wide range of heterogeneity in regard to the amount of refractive change and axial elongation alteration reported within these studies [17,18]. Comparing the findings also becomes difficult due to differences in both methodology and reporting protocols. For example, some studies rely on quantifying the “lighting experienced” via subjective assessment, while others rely on objective sensors. In addition, varying time points of assessments may influence results, especially when considering noted observed seasonal variations [40,41], but there may even be further differences based on whether assessments occur during weekdays or weekends, over academic periods versus vacation periods, or in children versus adults. In addition, defining “outdoor light exposure” by flat luminance thresholds, such as 1000 lux, has been debated [49,50], particularly as this level can be achieved in an indoor setting. Caution must be taken when interpreting the findings, with special attention to the nuanced differences in assessment and definitions of exposure. A more uniform standard of reporting and experimental protocols would be beneficial for future research efforts.

Further research examining the role of outdoor time across different stages of myopia also presents a complex picture. A prospective interventional study investigating a 30 min daily outdoor program over one year reported a reduction in axial elongation, but this effect was primarily observed in children who were non-myopic at baseline [51]. In children who were already myopic at baseline, the intervention did not show a significant effect on axial elongation [51]. Furthermore, the observed effect was not sustained after the program concluded [51]. Large-scale observational data reveals similar patterns. For instance, an analysis involving 835 myopic children found that time spent in outdoor/sports activities was not significantly associated with their annual rate of myopia progression [19]. These collective observations suggest that the relationship between habitual outdoor light exposure and myopia may differ depending on whether the focus is on initial onset or subsequent progression, highlighting an area needing further clarification to better understand the overall association between light and myopia.

**Figure 1:**
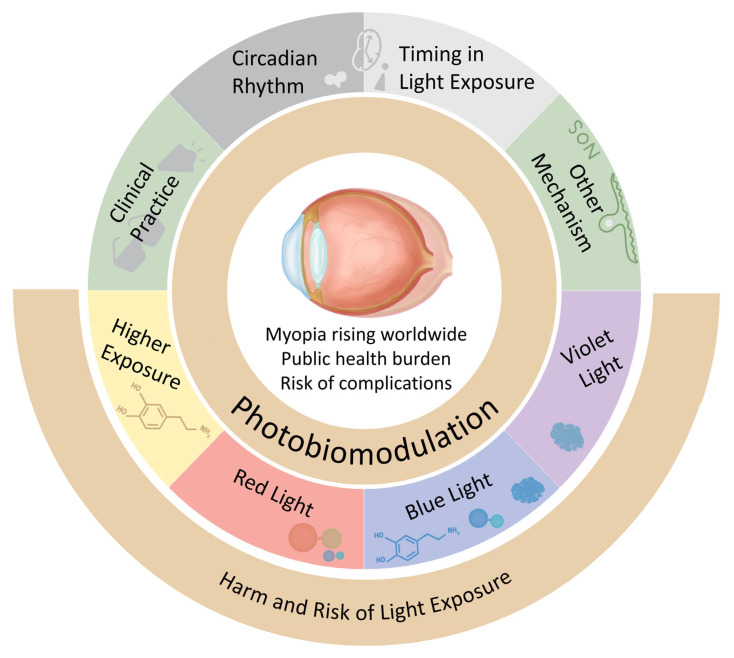
Graphical overview of photobiomodulation of myopia development. Photobiomodulation may influence ocular development through a variety of mechanistic pathways, but careful consideration needs to be taken when proposing intervention strategies.

Looking beyond primary outdoor interventions, complementary approaches and practical considerations are worth consideration. For example, evidence suggests that modifying indoor light environments may influence refractive development. A prospective randomized controlled intervention in primary schools in Foshan, China, compared standard classroom lighting with an “artificial natural light” intervention and reported a lower three-year cumulative incidence of myopia in the intervention group, alongside smaller myopic refractive shifts and reduced axial elongation relative to controls [52]. It may be that combining modifications of indoor environmental lighting with interventions increasing outdoor time may yield improved myopic prevention measures. From a practical perspective, the implementation of outdoor strategies may require balancing sun protection with adequate ocular illuminance. Measurements across common outdoor environments in Singapore demonstrated that illuminance levels outdoors remained far higher than indoors and commonly exceeded the threshold illuminance for myopia prevention (>1000 lux), even under shade, with hats, or with sunglasses, supporting the feasibility of combining sun-protective measures with outdoor-time recommendations [53]. Taken together, these findings suggest the substantial benefits of light exposure for refractive development, but also highlight our current lack of understanding of the full mechanisms at play. Due to this knowledge gap, it is difficult to define the potential causal relationship between myopia and light. In addition, to translate these benefits into controlled, evidence-based intervention strategies, the potential ocular risks associated with light must also be carefully considered—a focus that will be discussed in the following section. For a graphical illustrative overview of light’s influence on myopia, as well as factors that need to be taken into consideration when developing intervention strategies, see Figure 1. Over the following sections, this review aims to summarize some of the commonly proposed mechanisms by which light influences myopia, providing evidence from both animal and human studies, and outline current gaps in knowledge that will hopefully be addressed by future research efforts.

## 3. Light-Related Harm and Risks of Exposure

When considering the benefits of light exposure for ocular development, one must keep in mind the inherent dangers. While most proposed treatment strategies based on photobiomodulation are well within acceptable safety ranges, there are still some concerns about potential long-term consequences or adverse side effects. This is especially true since most treatment is aimed at school-aged children. The consequences of excessive light exposure or exposure to high-intensity or specific-wavelength light have been well documented. The International Commission on Non-Ionizing Radiation Protection lists six different types of ocular damage caused by visible and infrared radiation [54]. These include thermal damage to the cornea, iris, crystalline lens, and retina, as well as photochemical damage to the retina [54].

Blue light has been primarily reported to affect the cornea and retina, likely through oxidative stress as a key mechanism. Exposure of human corneal epithelial cells to blue light reduces their viability [55] and migratory capacity [56], accompanied by the production of reactive oxygen species. In mice, sustained blue light exposure delays corneal wound healing, shortens tear film breakup time, and ultimately exacerbates epithelial defects [56]. Studies in mouse models also demonstrate that illumination by blue LED induces S-opsin signaling collapse and rhodopsin translocation from the inner and outer segments to the outer nuclear layer, with this damage exhibiting an intensity-dependent effect [57]. Furthermore, electroretinography (ERG) a-waves and b-waves diminish with increasing irradiance, along with massive photoreceptor apoptosis in the central retina and an increase in 8-OHdG levels [58]. However, many of these studies utilize extreme scenarios which may not adequately reflect the typical environment. In fact, many clinical short-wavelength-based therapeutic trials for myopia control have demonstrated no severe complications [59,60,61]. There have also been suggestions that increased exposure to short-wavelength light emitted by backlit electronic devices (including computers, tablets, and smartphones) may actually increase myopia risk. However, most reviews have found little to no evidence of a causal relationship [62,63].

Red light injury reports are frequently associated with laser pointer injuries, mostly affecting the macula. The injury depends upon the wavelength, duration of exposure, intensity, and power density [64]. In a reported pediatric case, after multiple repeated sessions of shining a handheld red laser pointer into his eyes, a patient’s vision was reduced to counting fingers in both eyes, with fundus examination revealing yellow, striae-like lesions radiating from the fovea [65]. Optical coherence tomography (OCT) showed disruption of the outer retina, ranging from the outer plexiform layer to the retinal pigment epithelium [65]. Both visual acuity and OCT findings showed gradual recovery during a four-year follow-up period [65]. In one adult case study, accidental exposure to a red laser pointer resulted in a central scotoma and blurred vision [66]. Fundus examination revealed a yellow foveal lesion, while autofluorescence imaging demonstrated background hyperfluorescence with central hypofluorescence [66]. OCT displayed characteristic involvement of the outer retinal layers. Although the lesion size decreased over time, the patient reported persistent scotoma [66].

Infrared radiation has been associated with a significant increase in aphakia and all types of cataracts (subcapsular, cuneiform, and nuclear) among workers with occupational exposure [67]. This effect is supported by experiments in rabbits, where infrared exposure induced changes in the molecular weight and protein scaffold structure of the lens in an exposure-time-dependent manner [68]. Furthermore, the thermal damage effect of near-infrared radiation has been repeatedly reported to cause retinal damage, and damage thresholds have been established to increase safety [69].

While long-wavelength-treatment-based studies have frequently asserted its safety [70,71], there has been recent scrutiny that has led to some doubt [72]. In one case, after 5 months of red light therapy a 12-year old patient suffered bilateral vision loss, with bilateral foveal photoreceptor and RPE damage [73], and other cases have noted decreases in foveal cone density [74]. Study protocols have also been criticized for inadequate methods of assessing adverse events [72], which may have led to underreporting. Additionally, a recent study also found that just 3 min of use of two common red light therapy devices approached or exceeded the maximum permissible exposure as defined by the American National Standards Institute [75]. However, recent regulatory changes in the sale and manufacture of red light devices by the China National Medical Products Administration will hopefully reinforce safety protocols and ensure that all devices meet appropriate safety certification standards [76], while also encouraging other countries to adopt similar regulations.

Ultraviolet (UV) exposure is frequently implicated in ocular morbidity, and chronic exposure has been associated with a range of ocular diseases. Studies have shown a significant link between UV radiation exposure and the ocular conjunctiva, with the development of pterygium across diverse populations [77,78] Additionally, the risk of cutaneous melanoma is linked to pterygium presence and treatment history, raising suspicion of UV exposure involvement [79]. It has also be noted that periocular tumors arise on the lower lid and medial canthus at higher rates than the upper lid, indicating that these areas consistently receive proportionally more sun exposure are at greater risk [80]. Further reports indicate that various types of cataracts, including nuclear and posterior subcapsular cataracts, are also associated with higher ocular UV exposure [78,81]. The posterior segment of the eye may also be affected. An experiment involving young adults reported that in all subjects UV radiation at 315 nm was able to reach the retina, although the large inter-individual variability indicates that some people may be at greater risk than others for retinal UV damage [82]. As an acute, high-intensity example, a solar retinopathy study following ultraviolet radiation from sunlight described hyporeflective outer retinal defects, including ellipsoid and interdigitation zones, on OCT, with OCT angiography showing no corresponding choriocapillaris flow deficits [83]. In older adults, the Alienor Study estimated lifetime ambient UV radiation exposure and reported increased risk for cataract extraction and early age-related macular degeneration in both higher- and lower-exposure groups compared with intermediate exposure [84]. Notably, while epidemiological studies predominantly focus on adults, World Health Organization guidelines emphasize UV protection for children [85]. While two major violet light-based interventions in children reported no adverse outcomes linked to UV exposure [59,61], it is worth noting that the study participants’ daily overall UV exposure was typically less than 1 h per day. As such, there may be a need to investigate potential consequences of higher cumulative exposures.

Overall, these findings underscore the necessity to rigorously evaluate the efficacy and safety of light-based interventions when considering objective exposure metrics alongside clinically relevant ocular endpoints.

## 4. Circadian Rhythms

Circadian rhythms are endogenous oscillatory systems that evolved to adapt to the external light–dark cycle. Light functions not only as a physical stimulus but also as a primary environmental time cue that entrains these rhythms. Growing evidence suggests that ocular growth exhibits daily rhythmic variation rather than occurring uniformly across the day. Accordingly, circadian biology may provide an insight into the relationship between light exposure and refractive development.

Transmission of light-derived temporal signals may occur through the retina. In addition to its image-forming function, the retina detects light and influences sleep, mood, and homeostasis processes [86]. There is also communication between the retina and the suprachiasmatic nuclei of the hypothalamus. This exchange establishes photoentrainment, or the adjustment of the endogenous circadian clock to match the 24 h light–dark cycle [87].

Circadian rhythms have been shown to influence ocular growth and refractive development. Genetic interventions provide causal evidence for this. Retinal-specific knockout of the Bmal1 gene in mice resulted in myopic refractive shift and elongation of the vitreous chamber, consistent with axial components of common myopia [88]. In Drosophila models, knockout of cycle or period genes caused elongation of the optical pseudocone [88].

Beyond genetic evidence, observations of circadian rhythms in ocular biometric parameters in animals and humans also suggest that, instead of occurring uniformly and continuously, ocular growth is regulated by temporal dependence and visual input. Under normal visual conditions, chicks exhibit a diurnal pattern where axial length increases primarily during the day phase, while the choroid thins during the day and thickens significantly at night; retinal thickness remains largely stable [89,90]. This rhythmic response persists under constant darkness, supporting the existence of an endogenous circadian rhythm. However, this darkness-only rhythm differs from that observed under a normal light–dark cycle, producing effects similar to FDM model phenotypes [91]. Form deprivation enhances axial elongation and disrupts the normal diurnal organization of axial length, with the rhythm of axial length shifting to an advanced phase and choroidal thickness demonstrating larger amplitudes of rhythm compared to controls [90]. The development of this myopia is believed to stem primarily from a failure of the nighttime growth inhibition mechanism, rather than excessive daytime growth [92]. In humans, multiple studies have demonstrated that axial length in the human eye exhibits measurable diurnal fluctuations, typically on the order of 15–50 μm, with peak axial length occurring around midday, although the presence and magnitude of these rhythms vary across individuals and across days [93,94,95]. Both partial coherence interferometry and OCT studies have indicated that human choroidal thickness exhibits a diurnal rhythm characterized by nocturnal thickening and daytime thinning in antiphase with axial length [96,97,98]. This rhythm demonstrates good reproducibility and is associated with multiple factors, including axial length [98,99], refractive status [98,99], age [98], and systolic blood pressure [97]. Comprehensive imaging over a 24 h period under controlled light–dark conditions demonstrated that axial length, choroidal thickness, and multiple anterior and posterior segment parameters exhibit coordinated diurnal rhythms [99]. Amplitudes of axial length variations are in antiphase to choroidal thickness oscillations, and the amplitude of choroid variations is significantly correlated with axial length (*p* < 0.05) [99].

In summary, structural parameters closely associated with myopia progression, such as axial length and choroidal thickness, exhibit clear circadian rhythms. Therefore, circadian rhythms should not be overlooked and may serve as a temporal framework for understanding how light participates in the regulation of refractive development. However, long-term human studies directly linking light exposure, circadian alterations, and the onset or progression of myopia remain limited. As such, circadian disruption cannot yet be regarded as a definitive mechanistic explanation. The following section will further discuss other mechanisms supported by a stronger chain of evidence.

## 5. Light Exposure and Myopia Prevention: Potential Mechanisms

**Figure 2:**
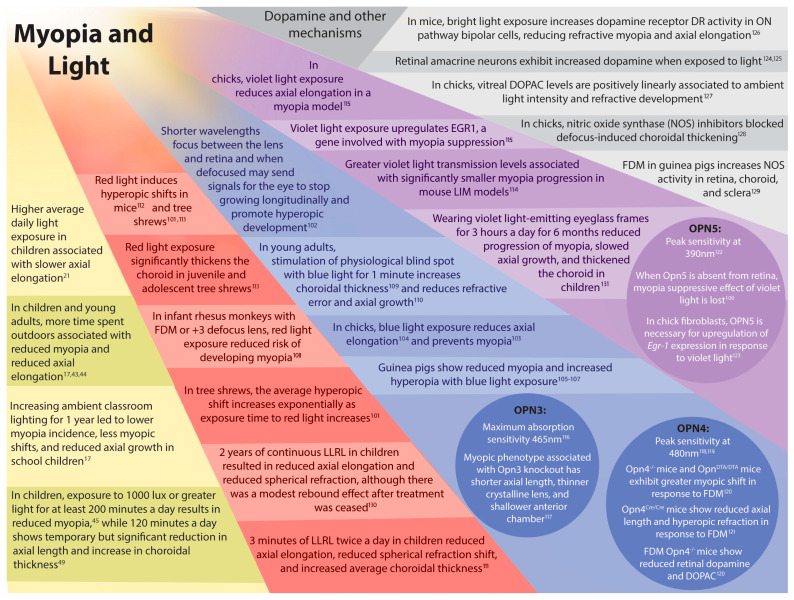
Visual summary of potential mechanisms and key supportive studies. FDM: form-deprivation myopia; LLRL: low-level red light therapy; DOPAC: 3,4-Dihydroxyphenylacetic Acid; EGR-1: early growth response-1 [17,21,43,44,45,49,100,101,102,103,104,105,106,107,108,109,110,111,112,113,114,115,116,117,118,119,120,121,122,123,124,125,126,127,128,129,130,131].

This framework provides a mechanistic basis for interpreting how lighting conditions, particularly the timing and regularity of the light–dark cycle, may influence ocular growth and refractive development. Studies investigating the relationship between light and myopia have led to several promising explanations, but no single theory has yet risen to prominence. The following section will discuss the various proposed mechanisms driving light’s influence on emmetropization. For a visual summary of proposed mechanisms and key study findings, please refer to Figure 2. There is a high probability that it is a combination of these mechanisms that are driving refractive development, and as such there is likely to be an overlap in findings. For example, light intensity is often related to dopamine release, and certain spectral wavelengths may trigger downstream cascades with diverse pathways. As such, the following subsections are not meant to provide singular explanations, but rather to propose factors capable of integration to bring about changes in ocular growth and refractive development.

In addition, when comparing these mechanisms, it becomes paramount to consider the fundamental differences in ocular physiology and spectral sensitivity between varying animal models. These differences may be one of the reasons studies often come up with varying, occasionally controversial results when comparing findings obtained in different species. For example, while short-wavelength light may result in delayed myopia onset in chicks [100,132] and mice [133], it appears that long-wavelength light conveys anti-myopic effects in tree shrews [134,135] and rhesus monkeys [136]. In addition, of the two common myopia models utilized in animals, FDM and LIM, it is still not certain which is a better representation of the human myopic condition.

These differences also emphasize why particular caution should be taken when attempting to translate animal study findings to humans. A previous report from the International Myopia Institute (IMI) has provided a detailed overview of the anatomical and physiological differences between common myopia animal models compared to humans [101]. Notable distinctions in photoreceptor types and distribution, ocular media transmittance, nonvisual opsins, visual acuity, and spectral sensitivity need to be considered when interpreting and comparing results.

For example, chickens have four types of single-cone photoreceptors [137] and one double-cone photoreceptor [138], while mice [139], guinea pigs [140], and tree shrews [141] are dichromats. Rhesus and cynomolgus monkeys are generally considered the animal model with the closest retinal physiology to humans, being trichromats with a rod-dominant retina [142,143]. Additionally, rhesus monkeys demonstrate an accommodative system similar to humans [144], which may play a role in the response to visually stimulated myopic development. Differences in photoreceptor distribution and spectral tuning can further differentiate results, and specific differences have been well described by another recent IMI report [145]. It has been well documented that chickens have different spectral sensitivities than humans, with much more sensitivity in the UV range [146]. Mice also appear to be particularly sensitive to UV light, with noted higher lens transmittance [147] and UV-sensitive cones [148]. This may be one of the reasons these animal models respond favorably to short-wavelength light-based treatments, while other animal models exhibit less robust associations.

Another factor worth considering when interpreting these studies is the variance in the spectral distribution of light sources. While many myopia-related studies report on the luminance values of “white light” used, this gives no indication of the spectral composition. Even natural sunlight’s spectral composition can vary over different seasons and at different points of day [102,149]. Artificial lighting typically presents a much narrower spectrum, but these spectral distributions can vary significantly between bulb types [145]. As such, experiments simply reporting on the effects of a “white light” source may miss the nuanced differences in photoreceptor activation across various species. Caution must be taken to not overgeneralize findings, especially when attempting to draw correlations between species. To advance light-based interventions for myopia control, future studies would benefit from more detailed reporting of both light intensity and the radiant power emitted at each wavelength.

In the following subsection, key animal studies and supporting findings from human studies are presented to help summarize our current knowledge of photobiomodulation and refractive development. It also aims to identify where our knowledge still falls short and highlight where translation of experimental findings to the human myopic condition remains particularly challenging.

### 5.1. Higher Light Intensities

One mechanism proposed early on was that it was simply the higher light intensities frequently present in outdoor light that drive myopia reduction. It was theorized that since children are spending more time indoors and indoor lighting levels are notably different than typical outdoor lighting intensities, this may explain the trend of increasing myopia development. For example, one study found that on a bright sunny day the mean light intensity was measured as high as 30,311 Lux, while the highest mean measure indoors (near a window with a source of bright sunlight) was only 4445 Lux [150]. Similarly, Bhandary et al. found that the median illuminance level in all outdoor locations tested was at least eight times higher than that of all the tested indoor locations [49]. Studies like these emphasized the disparity in illuminance levels, which then prompted studies to investigate the specific influence it may have on myopia development.

Most animal studies appeared to support this connection between light intensity and refractive development. Studies investigating the effect of rearing in dim light in chicks [151,152], guinea pigs [153], and rhesus monkeys [154] have demonstrated its ability to influence ocular growth. However, while rearing in dim light promoted myopic shifts in chicks [151,152] and guinea pigs [153], the opposite trend was observed in rhesus monkeys [154]. When considering FDM models, dim light does not influence the degree of myopia development in chicks [21], guinea pigs [153], or rhesus monkeys [155].

While rearing in dim light appeared to promote myopia development, rearing in high-intensity light induced more hyperopic shifts in most species studied. In tree shrews undergoing both LIM and FDM, moderately elevated light levels were able to slow myopia development in both models [33]. Chicks undergoing FDM reared in intense laboratory lighting of approximately 15,000 lux for 4 days demonstrated reduced axial elongation and less myopia than FDM chicks raised under normal lighting levels [21]. Similarly, rhesus monkeys with monocular FDM exposed to ambient lighting ranging from 18,000 to 28,000 lux, well within the normal range for what may be encountered on a sunny day outside, showed reduced axial elongation and reduced myopia development [24]. Studies in tree shrews [33], mice [156], and rhesus monkeys [24] have demonstrated that bright light exposure can provide protection against FDM. In some cases, high-intensity light even promoted hyperopic shifts in the untreated eye [24,33], although studies in chicks did not see hyperopic development in control eyes [21,23]. These variances may be due to study protocol differences such as treatment period lengths and the age of the animals used.

While the findings of animal studies have been encouraging and provided notable insight into the association between high-intensity light and myopia, there are still some conflicting findings between studies. These differences may be due to varying study methods, between-species differences in ocular physiology and spectral sensitivity, and variances in other visual cues within the rearing environment that could confound results. It can be challenging to translate these findings to possible applications in human children, and to date it has remained difficult to conclude that there is a direct causal role between light intensity and myopia protection in humans. However, several studies have generally supported a role for light intensity in ocular growth and development. In addition, recent efforts to promote reporting guidelines for light intervention studies in both animal [157] and human [158] studies may help improve future research and allow for more effective meta-analysis of findings.

In children, simply being exposed to light levels greater than or equal to 1000 lux for 200 min or longer during the day has been shown to result in less myopia [47]. Two trial studies similarly noted reduced myopia when increasing classroom illumination levels [159,160], with decreases in both refractive error shift and axial elongation, although there was some variation in the degree of change when considering subgroup analysis [160]. A cluster randomized trial in China also determined that the anti-myopic protective effect seen with increased outdoor time was related to the intensity of light experienced [161]. In another intervention, exposure to 500 lux or 1000 lux illumination for 120 min per day using a pair of light-emitting eyeglass frames resulted in a temporary but significant reduction in axial length and a significant increase in choroidal thickness, but this effect diminished 30 min after exposure [162].

Some studies investigated whether seasonal variations in ambient light exposure might also influence myopia development. One study found that having a larger average amount of daily exposure to lighting greater than 1000 lux was significantly associated with reduced axial elongation (*p* < 0.05), and moreover the change in axial length was significantly greater in myopes compared to emmetropes during the summer [163]. While emmetropes spent more time in outdoor light levels during the summer compared to the winter (*p* < 0.001), myopes exhibited no significant differences in the level of outdoor light exposure between summer and winter [163]. The differences in bright light exposure between summer and winter were also significantly associated with differences in axial elongation (*p* < 0.05) [163]. However, it is difficult to extract whether these differences may be at least in part due to potential behavior modifications due to myopia, for instance, being myopic may lead a child to prefer indoor near tasks as opposed to outdoor sports, or other observed temporal differences in natural sunlight.

When considering diurnal fluctuations in axial length and subfoveal choroidal thickness, myopes show significantly greater variations in axial length than emmetropes [164]. Moreover, these daily variations in axial length were negatively associated with daily bright light exposure time (*p* < 0.01) [164]. This indicates that more time spent in bright light might result in less fluctuation in the amplitudes of daily axial length changes, which in the long term may influence myopia development. To summarize, human studies have provided indirect evidence of the role of high-intensity light exposure in myopia development, but it is difficult to define a protective threshold intensity. Additionally, it is unclear whether high-intensity light is merely a proxy for outdoor time and that it is in fact a different component of outdoor light that is primarily responsible for the protection against myopia development. In addition to simply considering the intensity and amount of time spent in high-intensity light, research questions arose concerning whether it is a particular component of outdoor light that influences myopia. This led to a wave of spectral transmission studies, as discussed in the next subsection.

### 5.2. Spectral Pathways: Blue, Red, and Violet Spectrums as Potential Influencers of Myopia Development

**Figure 3:**
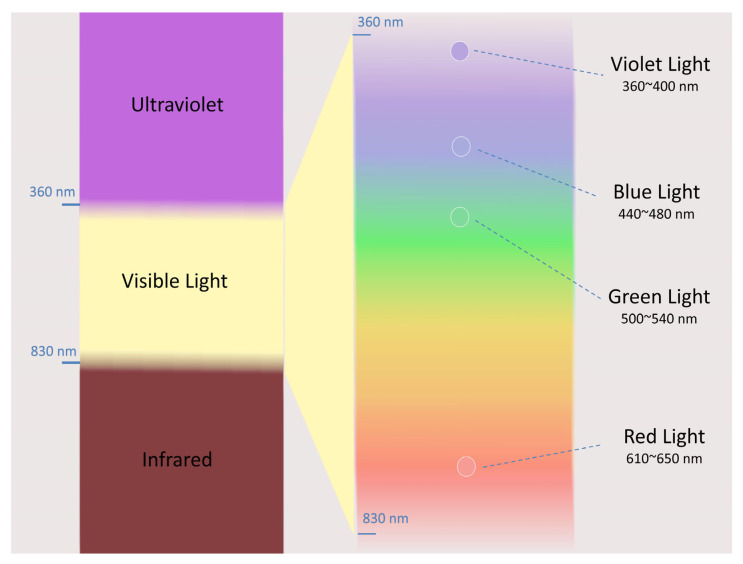
Spectrum range of visible and nonvisible light. Generalized wavelength ranges of ultraviolet, infrared, and visible light ranges for illustrative purposes.

In addition to just lighting intensity, researchers began to explore whether the spectral composition of outdoor light might be responsible for myopia prevention. See Figure 3 for an illustration of visible and nonvisible light ranges.

In addition to being generally dimmer than natural sunlight, indoor lighting consists of a different chromatic spectrum of light. Outdoor lighting consists of a higher concentration of shorter-wavelength light, such as blue and green wavelengths, rather than longer wavelengths like red [102]. There are also noted seasonal variations in the spectral distribution of natural sunlight. In the summer, short-wavelength blue light makes a significantly higher contribution to sunlight than in winter [102]. Dhakal et al. also found similar seasonal variations in spectral compositions, with significantly higher spectral power always exhibited in outdoor locations compared to indoor [165].

While short-wavelength blue light was one of the first spectrums proposed to prevent myopia, additional studies have demonstrated that short-wavelength violet light as well as long-wavelength red light may play a role. Currently, there is still no consensus over which spectrum has the most influence, and so a general summary of major studies is presented here regarding the respective wavelengths.

It still is not fully understood why there are conflicting and overlapping findings regarding which wavelengths provide protection against myopia development. One proposed theory for how differing wavelengths can modulate axial growth is the theory of longitudinal chromatic aberration (LCA). LCA is derived from the physical principles of light, namely, that short wavelengths have a shorter focal point than long wavelengths, and thus a blue or violet light will focus in front of the retina, while a red light will focus behind the retina. Based on this, if the red wavelength was in better focus, the eye would sense it was too long to focus blue wavelengths and thus slow axial growth. Conversely, if blue wavelengths are in better focus but red wavelengths are blurred, this indicates an eye that is too small and thus invokes axial growth. However, it is unknown whether the eye interprets stimuli in this fashion. Swiatczak et al. were able to potentially demonstrate this theory in action through their study published in 2022 [166]. Participants were presented with a film that was digitally filtered to either have blue wavelengths in sharp focus but red and green blurred, red wavelengths in sharp focus while blue and green were blurred, or an unfiltered control. When viewing the movie with the blue channel in focus, axial elongation significantly increased (*p* < 0.01) [166]. Conversely, when the red channel was in focus, there was significant axial length shortening compared to both the baseline and when the blue channel was in focus (*p* < 0.001) [166]. These findings were expanded upon when Gawne et al. demonstrated that tree shrews exhibited reduced axial growth and a hyperopic shift when the blue channel of a video display was isolated and blurred (*p* < 0.0001) [167]. Both the study by Swiatczak et al. and the study by Gawne et al. created defocus through the use of spatial frequency patterns and low-pass filters, rather than true optical defocus, which needs to be considered when making inferences from the findings. In fact, it is difficult to argue that LCA is the only mechanism influencing axial growth, especially in light of the fact that studies have found varying support for both blue light and red light as positively influencing myopia. It is more likely that there are a variety of complex mechanisms influenced by the entire visual spectrum. It may be that LCA only applies in particular settings, such as fine tuning when refraction is near emmetropia [168]. As noted by Seidemann and Schaeffel, there also may be several different cone types modulating emmetropization [169], which may in part explain why some species show preferred sensitivities to specific wavelengths. In another curious finding, in tree shrews a flickering short-wavelength blue light stimulus induced myopia, but a steady blue light promoted emmetropization [135]. This study highlights how there may be a range of defocus cues influencing the process of emmetropization. A downside to many of these studies, including some of those discussed below, is that they create an open-loop feedback system. In reality, emmetropization is more likely a closed-loop feedback system, where when a stimulus is presented the eye’s emmetropization mechanisms are engaged and balance the changes. In these open-loop experimental settings, the stimulus persists regardless of how the eye responds, and so the resulting phenotype may not accurately represent an outcome from a real-world setting. In addition, it is challenging to isolate the effects of defocus from color information, which can confound interpretations of whether the ocular responses are truly due to the spectral wavelengths or whether they may be due to differing color vision among species. There may also be species-specific sensitivities to particular wavelengths, as well as differing distributions of photoreceptors which can lead to varying findings.

Overall, more research is needed to determine the complex mechanisms driving these spectral responses. In the meantime, consideration of what is known about the ocular responses to varying wavelengths, including variations between species, may help point to the potential molecular processes at work and help develop the full picture.

#### 5.2.1. Blue Wavelength

Natural sunlight has a much higher composition of short-wavelength blue light than common indoor lighting sources [103]. It has been hypothesized that since in humans only about 7% of cone excitation is due to wavelengths shorter than 500 nm, there is a general lack of sensitivity to these wavelengths, and indoor lighting that is already deficient in short wavelengths cannot provide the stimulation necessary to adequately promote emmetropization [104]. According to the theory of LCA, shorter wavelengths focus between the lens and retina and when defocused may send signals for the eye to stop growing longitudinally and promote hyperopic development [166]. Experimental animal models have generally supported these hypotheses for blue light’s positive influence on refractive development.

The trend of blue light preventing myopia progression was first identified in chicks nearly two decades ago. In chicks, blue light exposure results in significantly less myopic shift [132] and reduced axial elongation [170] in FDM models. Chicks reared in red light show a trend of increased axial length compared to those reared in blue light [171]. Blue light exposure induces hyperopia during the rearing period, as well as the ability to reverse red light-induced myopia [171]. Similarly, compared to white light, chicks reared in 615 nm light are significantly more myopic than those reared in 430 nm light (*p* < 0.01), and if chicks raised in 615 nm light are transferred to 430 nm light, the trend reverses and chicks become more hyperopic (*p* < 0.05) [169]. When chicks are raised in orange light, they once again demonstrate a significantly larger shift in lens-induced myopia and altered ERG responses compared to chicks raised in blue or white light [105]. Since chick eyes are excellent at transmitting short-wavelength light, this may be one of the reasons they appear particularly sensitive to blue and violet light treatments [106]. However, similar trends have been observed in several other animal models.

Guinea pigs have shown a similar reduction in myopia and increased hyperopia when exposed to short-wavelength light [107,108,172]. In addition, in an LIM model of myopia in guinea pigs, blue light exposure increased choroidal thickness compared to white light (*p* < 0.01), while red light exposure temporarily thinned the choroid (*p* < 0.01) [109]. While blue light exposure was able to suppress both myopia and axial elongation, red light had no significant effect on myopia development within the LIM model [109].

In one study of infant rhesus monkeys, there was no development of myopia in those raised under 455 nm blue light, but two out of nine monkeys raised in red light developed myopia and demonstrated a faster increase in vitreous chamber depth [110]. This led the researchers to suggest that monochromatic red light may be a risk factor for myopia development, but when the two myopic animals were excluded there were no significant differences in refraction between those reared in blue, red, or white light [110]. In fact, other studies to be discussed later have found that red light actually protects against myopia in rhesus monkeys. As such, it may be that for rhesus monkeys there are individual differences in sensitivities to particular wavelengths of light but not a species-wide trend. Alternatively, it may be that light exposure during different development periods or for different durations may lead to different phenotypes, but more research is needed.

In young adults, varying periods of blue light exposure have demonstrated encouraging findings for myopia prevention. One hour of blue light exposure over a period of four days was able to reduce the axial length of both a focused and 3-diopter hyperopic defocused eye, while red light exposure increased the axial length from baseline and decreased the choroidal thickness [103]. Two relatively recent studies have also demonstrated that stimulation of the physiological blind spot, or optic nerve head, with blue light for 1 min results in increased choroidal thickness [173] and a significant interaction between axial length and refractive error changes [174]. However, it is worth noting that one study found that it is only retinal stimulation with blue light, not stimulation of the optic disc, that could potentially convey anti-myopic influences [111]. These differences may be explained by methodological differences, such as stimulating monocularly versus binocularly and the different light sources used, but they do indicate a need for more studies to consistently confirm the findings. The mechanism by which blue light influences myopia is not well defined. Animal studies have indicated that nonvisual opsins, discussed later, may in part be responsible, but this has yet to be confirmed in humans.

While there is a notable amount of evidence supporting blue light interventions in both animal and human studies, there has also been a body of evidence presented to support long-wavelength red light as an influencer of myopia development. In addition, there remain several unanswered questions, such as why there are widely differing results when comparing different species. These variances make it challenging to fully translate blue light interventions to humans, and more research is needed to understand the mechanisms driving the associated ocular changes.

#### 5.2.2. Red Wavelength

Red light consists of longer-wavelength light falling between 600 and 700 nm. According to most theories of emmetropization feedback, including the LCA theory, red light focuses behind the retina, which should stimulate axial elongation and result in the promotion of myopia [101,112]. However, somewhat paradoxically, many animal and human studies have shown the opposite effect, with red light promoting hyperopia and slowing axial elongation. For this reason, red light’s influence on myopia cannot be explained by classic emmetropization theories, and its full mechanism remains to be elucidated. All the same, there is a strong body of research to support red light’s protective role against myopia development, even if its mechanisms remain unclear.

Red light induces hyperopic shifts in tree shrews [134,175], and the average hyperopic shift increases exponentially as exposure time increases [175]. In both juvenile and adolescent tree shrews, red light exposure significantly thickened the choroid and reduced vitreous chamber depth compared to controls (*p* < 0.05) [134]. In infant rhesus monkeys, red light exposure reduced the risk of developing myopia in both FDM models and those with a +3 D defocus lens [110]. In another study, infant rhesus monkeys wearing long-wavelength-pass filters demonstrated a significant hyperopic shift compared to those wearing neutral density filters (*p* < 0.001) and normal controls (*p* < 0.001) [176]. However, after removal of the long-wavelength-pass filters, the induced hyperopia dissipated [176]. In mice, red light has been shown to induce hyperopic shifts [113], but in contrast short-wavelength light, especially violet light, has been shown to confer protection against experimental myopic development [114,133,177]. While short-wavelength light’s influence could potentially be explained by the involvement of nonvisual opsins, long-wavelength light seems to influence ocular growth through a separate pathway, which may account for some of the differing results.

While it is unclear how red light influences ocular growth, one at least partial mechanism that has been proposed is the nitric oxide (NO) system [115]. While red light has been shown to compensate for reduced NO and therefore reduce oxidative stress [178], this still has yet to be fully explored in an ocular context. It has also been demonstrated that red light can influence metabolic processes in the retina [179] and sclera [180], which may modulate ocular growth. More studies are needed to confirm the mechanism of red light therapy, but there do appear to be positive findings in several studies to suggest its benefit.

Overall, these preliminary studies have provided encouraging findings, and the addition of upcoming results of a United States clinical trial may pave the way for a new therapeutic option for myopia control based on long-wavelength light.

#### 5.2.3. Violet Wavelength

Of the proposed wavelengths involved in myopia control, violet light is the newest to enter the field. Violet light belongs to the visible light range of 360 to 400 nm wavelength, and while it technically falls within the UVA radiation spectrum as defined by the World Health Organization (315 to 400 nm) [85], there is still some debate about its risk potential. As several studies have demonstrated its link to myopia control with no serious adverse outcomes observed, it is quite possible that it may be used safely under appropriately controlled conditions.

In chicks, violet light exposure not only reduces axial elongation in a myopia model, but it upregulates the gene Early Growth Response 1 (EGR1), which is known to be involved in myopia suppression [100]. In fact, violet light was significantly better at inducing upregulation of EGR1 in chick chorioretina compared to blue light exposure [100]. In mice, violet light exposure has been shown to inhibit myopia development and progression in LIM models [114,133,177]. There also appears to be a dose-dependent relationship between the amount of violet light transmission and myopia progression, with greater transmission levels being associated with significantly smaller myopia progression in LIM models compared to controls [177]. In addition, one study demonstrated that when comparing violet, blue, green, and red light exposure, violet light produced the most significant reduction in refractive change and axial elongation in LIM mice [133].

While there have only been a handful of studies examining the effect of violet light on myopia progression in humans, the results have been moderately encouraging. A retrospective study comparing children who wore violet light-blocking eyeglasses, partially blocking contact lenses, or violet light-transmitting contact lenses found that those who wore violet light-transmitting contacts had the greatest myopia suppression effect [100]. In another retrospective study comparing adults who received non-violet light-transmitting phakic intraocular lenses (IOLs) and others who received violet light-transmitting phakic IOLs, over a 5-year period those with non-violet light-transmitting phakic IOLs had nearly double the amount of myopic change and four times longer changes in axial growth [181]. It is important to note, however, that this study did not take into account factors such as differences in time spent outdoors, amount of near work, and parental myopia status.

The mechanism driving violet light’s influence on ocular development is still not wholly elucidated, but evidence from several animal studies has suggested a role of nonvisual opsins, as discussed in greater detail in the next section. Still, there is a lack of studies attempting to investigate the direct mechanism in humans, and intervention trials with violet light have yielded varied findings [59,61,100,182]. As such, the question of how influential violet light may be in human myopia development remains unanswered.

As violet light has only recently begun to attract attention as a possible means of preventing myopia, it does not boast the robust evidence base that blue and red wavelength treatments have accumulated. However, future research may continue to build upon the base that has been established and may provide substantial and consistent results to confirm its role.

### 5.3. Nonvisual Opsins

Since light exposure, regardless of the specific spectrum, has been linked to myopia prevention, this has led to expanded exploration of the opsin family of G-protein-coupled receptors (GPCRs), specifically the nonvisual opsins, also known as noncanonical opsins. Evolution has led mammals to possess a diverse array of GPCRs to detect light, and these light-sensitive receptor proteins are called opsins. Some opsins, such as the rod (OPN2) and cone (OPN1SW, OPN1MW, and OPN1LW) opsins, are related to visual function and have been extensively explored. However, of the nonvisual opsins encephalopsin (OPN3), melanopsin (OPN4), and neuropsin (OPN5) much less is known. Recently, the nonvisual opsins have been garnering attention as potentially playing a role in myopia development. Here, we provide a brief review of each of these opsins and outline the potential mechanisms by which they influence myopia development.

#### 5.3.1. OPN3 (Encephalopsin)

Despite the fact that OPN3 was the first of the nonvisual opsins to be discovered, with its first identification over 25 years ago in the adult mouse brain [183], its full function is still not understood. It has only recently been identified as potentially playing a role in myopia development, and much more investigation is necessary. Opn3 is abundantly expressed throughout the body, and it has been identified in the brain [183], heart [184], lungs [185], eyes [186], skin [187], adipose tissue [116], and immune system [188]. OPN3, along with OPN4, shows expression in the mouse eye as early as E11.5, while traditional rod and cone opsins are usually not expressed until post-natal development [186]. OPN3 is particularly sensitive to blue light, with a maximum absorption sensitivity of approximately 465 nm [189], and thus it may be part of the pathway relating blue light to myopia prevention. In addition to its light-dependent functions, OPN3 exhibits light-independent functions such as muscle relaxation [190], metabolism [191], and immunological processes [188]. Since OPN3 is present in the epidermis, studies have investigated its role in wound healing. For example, exposure to blue light stimulates wound closure with a corresponding increase in OPN3 expression, and OPN3 may be required for maintaining epidermal barrier function [117]. A lack of Opn3 does not appear to result in any major behavioral, learning, memory, or motor coordination issues in mice, but Opn3-deficient mice do demonstrate an attenuated acoustic startle reflex, although it does not seem to be dependent on light in any way [192].

In regard to its potential role in myopia development, much less is known about OPN3. Opn3 is expressed in a small subset of retinal ganglion cells and choroidal cells [193]. In a recent study utilizing Opn3-phiC31o mice to knock out Opn3 function, the Opn3 knockout mice demonstrated myopic refractions compared to wild-type controls [194]. However, in a study of the weekly refractive development of both retina-specific and germline Opn3 knockout mice exposed to an LIM model, it was only the germline knockout which exhibited a mildly myopic phenotype [193]. This myopic phenotype was also somewhat atypical, with a shorter axial length, thinner crystalline lens, and shallower anterior chamber [193]. The loss of Opn3 function does not prevent myopia development via LIM, but it does appear to change the resulting phenotype [193]. Studies in both FDM [195] and LIM models [118] have elucidated a gene signature associated with induced myopia development known as GO/GROW. Opn3 null mice show a distinct change in the regulation of a small subset of GO/GROW signature genes, indicating that OPN3 activity may be responsible for expression changes in response to refractive mismatch in some GO/GROW signature genes [193]. In summary, while OPN3 is abundantly expressed throughout the body and may have a role in wound healing, metabolism, and immunological processes, there is a lack of studies specifically on OPN3’s involvement in myopia development, and much more investigation is warranted to elucidate its potential role.

#### 5.3.2. OPN4 (Melanopsin)

Compared to OPN3 and OPN5, OPN4 has received notable research attention over the past few decades, although relatively few studies have concentrated specifically on its potential contribution to myopia development. It was first identified in 1998, in the hypothalamus, retina, and iris of Zenopus laevis [119]. Research has demonstrated its vital role in the regulation of circadian rhythms, although there is some variation between vertebrate species [196]. The peak spectral sensitivity of OPN4 is in the blue light range, at approximately 480 nm [120,121], although differences in measurement methods and species result in some variation [122]. Regardless, this peak sensitivity suggests that OPN4 may play a role in the relationship between blue light and refractive development, although it has yet to be fully described.

Concerning myopia development, there have been two main studies investigating OPN4’s potential role, the results of which are conflicting. A study by Chakraborty et al. studied OPN4’s response to FDM using Opn4^DTA/DTA^ mice, which have neither intrinsic nor photoreceptor-mediated melanopsin-expressing retinal ganglion cell responses, and Opn4^−/−^ mice, which lack functional melanopsin photopigments and intrinsic mRGC responses but maintain input from photoreceptor-mediated RGCs [197]. Compared to wild-type controls, both Opn4^−/−^ mice and Opn4^DTA/DTA^ mice exhibited a greater myopic shift in response to FDM [197]. In addition, when experiencing FDM, retinal dopamine and 3,4-Dihydroxyphenylacetic Acid (DOPAC) were reduced by approximately 20% in Opn4^−/−^ mice [197]. Opn4^DTA/DTA^ mice, alternatively, showed no alteration in dopamine or DOPAC levels when exposed to FDM [197]. Treating the Opn4^−/−^ mice with systemic L-DOPA resulted in a reduction in the induced myopia. The results overall indicate that disrupting retinal melanopsin signaling affects refractive development and increases susceptibility to FDM through a dopamine-dependent mechanism [197]. Dopamine, discussed in greater detail in a later section, has often been raised as an explanatory pathway connecting light to ocular growth and refractive development. Studies like those by Chakraborty indicate that OPN4 may be a part of this pathway, but this has yet to be confirmed by further research.

In fact, a study by Liu et al. came to different conclusions regarding OPN4 and refractive development. When selectively ablating ipRGCs in mice using melanopsin–saporin (MEL-SAP), an immunotoxin, there was an attenuated myopic shift in response to FDM compared to control mice [198]. In addition, Opn4^Cre/Cre^ mice exhibited reduced axial length and hyperopic refraction in response to FDM compared to wild-type controls [198]. MEL-SAP-treated mice should essentially be analogous to the Opn4^DTA/DTA^ mice utilized in the Chakraborty study, but the results were remarkably different. Some of these differences may be due to methodological differences and different protocols when determining control groups.

OPN4 is well known to play a role in the regulation of circadian rhythms, but the mechanism by which it influences myopia is still not understood and whether it promotes or prevents myopia progression still remains to be determined.

#### 5.3.3. OPN5 (Neuropsin)

OPN5 has been located within the retina, brain, and outer ears of mice, and is responsible for activating heterotrimeric G protein Gi when exposed to particular wavelengths of light [199]. Its peak sensitivity of 380 nm is the same in both mice and humans [199], although there is a suggestion that mice may be more sensitive to this wavelength [148]. This peak wavelength, within the violet light range, is considered part of the UVA radiation spectrum as defined by the World Health Organization (WHO) [85]. As such, it is generally considered a high-risk wavelength, and most modern filters on windows and lenses block this spectrum. While abundant in natural outdoor sunlight, modern indoor lighting notably lacks violet light [200], so while OPN3 and OPN4 may be activated indoors, it is less likely for OPN5 to be activated.

Disruption of OPN5 results in erratic light-dependent vascular development within mice postnatal eyes, and this is thought to be due to violet light exposure increasing activity of the dopamine transporter via OPN5 and vesicular GABA/glycine transporter [201]. Retinal dopamine appears to be modulated in part by violet light exposure, and when OPN5 function is disrupted there is a corresponding decrease in retinal dopamine levels [201]. In chicks, studies have similarly demonstrated that exposure to blue and violet light promotes increased retinal dopamine release [132]. Stimulation with 480 nm blue light in guinea pigs results in a similar increase in retinal melanopsin mRNA, which modulates dopamine signaling, as well as resulting in a less myopic phenotype [108]. Numerous studies have implicated dopamine’s role in myopia development [202,203], and its potential mechanistic pathway will be discussed in greater detail in a later section. In both FDM and LIM models, the resulting reduced retinal dopamine is generally considered a cue to promote myopic axial elongation [204]. While retinal dopamine level modulation has previously been attributed exclusively to OPN2 [205], it has since been demonstrated that OPN5 [201,206] and OPN4 [197] are implicated in dopamine regulation. It is possible that the mechanism through which OPN5 influences myopia development involves a dopamine-dependent pathway, but this has yet to be confirmed.

Stimulation of Opn5 RGCs with violet light in LIM mice prevents the induced myopic shift [133]. Additionally, when comparing white light supplemented with blue (440–480 nm), red (610–650 nm), green (500–540 nm), or violet light (360–500 nm), violet light supplementation provided the strongest suppression of myopia development in an LIM mouse model [133]. When Opn5 is specifically deleted from the retina using Chx10-Cre;Opn5^fl/fl^ mice, the myopia suppressive effect seen with violet light exposure is lost [133]. These mice also demonstrate notably thinned choroids [133], indicating that OPN5 may be in part responsible for modulating violet light’s influence on choroidal thickness. In addition, OPN5 appears to play a role in the expression of early growth response-1 (EGR-1) in the retina [207]. EGR-1 is included in the aforementioned GO/GROW signature genes, and it is integral to both refractive development and ocular growth [123,124,208]. While violet light exposure elicits an increase in retinal Egr-1 mRNA expression in wild-type mice, this increase in expression is absent in Opn5 knockout mice [207]. Similarly, it has been shown in chick fibroblasts that OPN5 is necessary for the upregulation of Egr-1 expression in response to violet (375 nm) light exposure [125]. It has also been suggested that retinal dopamine may be involved in the violet light-dependent OPN5-EGR-1 pathway [201,207], but this remains to be fully confirmed. EGR-1 is well known to be modulated by a range of stimuli, so it may only be one of several potential downstream mediators, and it is unlikely that its regulation relies solely on OPN5 stimulation. However, much of the research related to OPN5 and myopia development supports the hypothesis that the lack of violet light wavelengths in our modern environments has helped promote the increased prevalence of myopia over the past several decades, and further investigation into its role is warranted.

Overall, OPN5 has been implicated in light-dependent vascular development, dopamine modulation, and the expression of GO/GROW signature genes that may influence myopia progression, with several animal models collaborating real-world hypotheses, but more definite studies are needed to specifically identify downstream mediators and confirm whether there is a dopamine-dependent pathway.

### 5.4. Dopamine and Other Molecular Influences

The molecular mechanisms behind light’s influence on refractive development are still not fully understood, but great strides have been made in recent years in elucidating potential pathways. One recurring player in the light and myopia story is dopamine, as briefly mentioned in prior sections. Dopamine, a neurotransmitter, is highly involved in a great number of central nervous system functions, both directly and indirectly [209]. It was first linked to myopia over 30 years ago when Stone et al. demonstrated that chicks with myopia induced by FDM had reduced levels of dopamine as well as its metabolite DOPAC [202].

Retinal amacrine neurons exhibit increased dopamine formation when exposed to light [210,211] and activate various cellular pathways incorporating cAMP- and cGMP-dependent protein kinases [212]. As summarized by Bloomfield and Völgyi, dopamine likely modulates the conduction of gap junctions between rods and cones via activation of D2/D4 receptors [213]. There is extensive coupling between cones, between rods and cones, between rods and cones and bipolar cells, and between rods and cones to amacrine and horizontal cells [213]. This coupling appears to be to some extent modulated by circadian rhythms, with dopamine release leading to reduced conductance between rods and cones. However, at night, when light levels are typically low, dopamine release is reduced and the coupling between rods and cones increases, allowing for better visual detection in dim conditions [213]. Dopamine also acts upon varying cell types to modify receptive field sizes, which are known to adapt dynamically to changing visual environments [214]. It is possible that one of the mechanisms by which dopamine influences myopia development involves this retinal fine tuning. For example, dopamine might promote a reduced retinal receptive field size, creating less perceived blur and reducing the inclination to extend the axial length. When chicks [215,216], guinea pigs [217], mice [218], and tree shrews [215] experience prolonged retinal defocus, there is a noted decrease in retinal dopamine levels with the induced myopia. In chicks, when partially occluding goggles were applied, only the retinal areas where myopia was induced demonstrated reduced DOPAC [219,220]. Axial growth, therefore, seems to be inversely related to dopamine levels in the retina.

Dopamine is frequently referred to as a “stop signal” for myopic growth, and its release in response to light modulates eye growth via distinct dopamine receptors [204,221]. It is worth mentioning that some studies have indicated that FDM does not appear to alter retinal dopamine levels or DOPAC in wild-type mice [222,223,224], indicating that there may be differences in emmetropization processes between species and due to myopia induction model choice, but in general most species support the finding of an association between dopamine and myopia. Increasing retinal dopamine through either direct ocular injection or L-DOPA treatment has been shown to prevent FDM in rabbits [225], guinea pigs [126], and mice [226]. In chicks, Karouta et al. recently demonstrated that bright light inhibits LIM development and is at least partly dependent upon a dopamine-dependent mechanism [127]. In mice, bright light increases dopamine receptor DR activity in the ON pathway bipolar cells, resulting in reduced refractive myopia and reduced axial elongation [156]. While Landis et al. found that myopia susceptibility to LIM in mice was reduced in both scotopic and bright photopic settings compared to mice housed in mesopic lighting, the highest dopamine activity was noted within the photopic group [227]. In fact, vitreal DOPAC concentrations in young chicks appear to be positively linearly associated with the intensity of ambient light, and refractive development, in turn, is strongly associated with these levels [228]. In chickens, periods of high illuminance (15,000 lux) were able to partially recover retinal dopamine release, as measured by vitreal DOPAC, in an FDM model [229].

While the connection between dopamine and myopia in humans has yet to be definitively described, it has been observed that in children prescribed methylphenidate hydrochloride (MPH), a dopamine uptake inhibitor, for attention deficit hyperactivity disorder, there was a lower incidence of myopia [230] and that myopia progression was slowed [231]. MPH has also been shown to suppress experimentally induced myopia in chicks, likely through increasing retinal dopamine synthesis [232].

Findings such as these reinforce the idea that light may influence myopia development via a dopamine-dependent mechanism, but this is likely not the only pathway. In all likelihood, the dopamine mechanism involves additional molecular signals. For example, ocular all-trans retinoic acid (atRA) has been reported to vary with the duration of exposure to different ambient lighting, and retinal atRA levels were observed to decrease with increased dopamine, indicating that their interaction may modulate myopia development [233]. In addition, it is important to note that dopamine levels are reduced in both LIM and lens-induced hyperopia models [234], but the axial growth modulation occurs in different directions. This indicates that dopamine may act more as a signal for modulating ocular growth [127] and that it is other downstream factors that dictate the refractive shifts.

Several studies have implicated nitric oxide (NO) as yet another player in the ocular growth signal cascade [235,236]. Studies have shown that NO release can be stimulated by ultraviolet light [237], blue light [128], and red light [129,238]. In chicks, nitric oxide synthase (NOS) inhibitors were reported to block defocus-induced choroidal thickening, supporting a role for NO-related signaling in visually driven choroidal responses [239]. FDM in guinea pigs results in increased NOS activity in retinal tissues [236]. In a separate study, FDM induced increased NOS activity in the retina, choroid, and sclera in guinea pigs [240]. These changes in NO and NOS influence cyclic GMP (cGMP) concentrations, and it has been suggested that cGMP concentrations may mediate NOS activity [240]. Increasing cGMP experimentally through pharmaceutical interventions prevents fibrosis by inhibiting myofibroblast differentiation, as demonstrated in the skin [241] and kidney [242,243]. For this reason, cGMP levels may influence myopia through modification of scleral fibroblasts. NO appears to inhibit myopia in a dose-dependent manner [235], but the specific mechanisms behind its influence have yet to be described.

Melatonin has also been connected with myopia, with suggestions that endogenous melatonin levels may promote myopia progression. If this is the case, circadian entrainment and sleep–wake patterns may influence refractive development. Light exposure modulates systemic melatonin levels [108,244,245], which sends signals to indicate whether it is daytime or nighttime. Overall, studies on melatonin’s relation to myopia have been conflicting. In some studies, myopes demonstrate lower melatonin production, delayed sleep onset, and more sleep disruptions [246,247], but it is unclear whether this is directly related to circadian phases [248]. Alternatively, other studies have found that myopes demonstrate elevated levels of melatonin [248,249,250]. A study by Burfield et al., meanwhile, found no notable difference in systemic melatonin levels between emmetropes and myopes [95]. Similarly, while light exposure and time outdoors appear to influence morning melatonin concentrations, there is no notable difference in levels based on refractive status [251]. These findings indicate that melatonin’s role in myopia development, if existent, is complex and remains to be discovered.

## 6. Importance of Timing in Light Exposure and Myopia Development

Light exposure varies throughout the day, making timing a critical dimension alongside intensity and duration. Light timing may influence myopia-related outcomes such as axial elongation and refractive error, and exploring its influence complements existing research perspectives.

Animal studies have shown that mistimed light exposure can disrupt intrinsic physiological rhythms and interfere with normal ocular development. In chicks, normal emmetropization requires a regular light–dark cycle, with at least 4 h of light and darkness daily [35]. Brief nocturnal light exposure has been shown to disrupt circadian rhythms, alter axial length, and induce choroidal thickness changes, implicating circadian disruption induced by abnormal lighting conditions as a pathway toward ametropia [252].

Nighttime light exposure is a common disturbance in modern life and may serve as a supplementary factor in human studies. Early observational research did not support a strong association between nighttime light exposure during infancy or early childhood and the presence of myopia in adulthood [253]. However, recent cross-sectional studies involving school-aged children reported that higher nighttime light exposure indicators are associated with increased myopia prevalence [254,255]. Researchers have even proposed that a “nighttime healthy light” environment may serve as a potential strategy for myopia prevention [255]. Although confounding factors exist and robust evidence-based findings have yet to be supplied, this perspective offers valuable insights and inspiration for how light exposure timing may affect ocular growth.

Animal studies also reveal that the effect of light exposure depends on the precise timing of intervention and the baseline refractive state of the eye. For example, in chick models, applying short-term intense light exposure at different times to eyes in varying states of FDM produces varying effects on axial length elongation. For myopic or hyperopic defocus eyes, evening light exposure more effectively inhibits axial elongation, whereas morning light has no effect [256]. For untreated normal eyes, morning light stimulates axial length growth [256]. However, when combined with hyperopic defocus, morning light exhibits a stronger inhibitory effect [256]. A similar study comparing 2 h windows of 10,000 lux at morning, midday, and evening for FDM chicks found that midday exposure was the most effective at inhibiting myopia [257].

Light intervention at specific wavelengths also exhibits a “time-window” effect. For instance, in a mouse study involving violet light, Jiang et al. tested multiple intervention time points and discovered that the period with the best myopia-suppressing effects occurred around dusk [133]. These findings indicate that the timing of light exposure influences the extent of ocular growth modulation. While these findings are limited, they hint at a complex relationship where the effectiveness of light exposure may be modulated by the time of day. Interpreting study findings may be further complicated by differences in endogenous circadian signals and species-specific behavioral patterns (such as being diurnal versus nocturnal), and much more research is necessary to fully describe temporal variances in effectiveness.

Evidence regarding the timing of light exposure in humans is still awaiting broader and more in-depth research. One study indicated that in healthy adults, short-term enhancement of morning light exposure via light-emitting glasses can lead to an increase in subfoveal choroidal thickness [130]. However, measurements at other times of day were not taken, so it is difficult to confirm if this effect shows variation based on application period. Another recent study demonstrated that moderating light intensity during sleep and just prior to waking may contribute to myopia prevention [255]. Overall, there is still a notable lack of randomized controlled evidence directly comparing light interventions across different time windows. Current evidence primarily consists of short-term physiological studies, as mentioned in the previous section on circadian rhythms.

Overall, the existing literature suggests that the timing of light exposure, including its distribution throughout the day and the nocturnal light environment, may influence axial elongation and myopia progression. This implies that future light-related myopia research and interventions should consider not only light intensity and duration, but also the timing of light exposure. To advance this field, future studies should compare the intervention effects of light administered during different time windows while controlling for light intensity and spectral composition. For example, it may be that particular wavelength-specific treatments, such as red light or violet light therapies, may show vastly different efficacies based on whether applied in the morning, at midday, or in the evening before bed. Subsequent side effects may also need to be considered, such as whether treating at particular times may maximize myopia prevention but at the cost of disrupting circadian sleep–wake cycles. Practically, there also need to be more studies to determine what constitutes an effective treatment duration, as this, too, may influence when treatment is best administered. From a broader perspective, incorporating this into the research framework also opens new avenues for integrating myopia research with circadian rhythm and behavioral studies.

At the same time, when interpreting findings from relevant animal studies, it is crucial to fully acknowledge the differences in behavioral rhythms and physiological regulatory mechanisms between diurnal and nocturnal species, avoiding the simplistic extrapolation of animal experimental results to human applications. Particularly in humans, light exposure is often influenced by a combination of artificial lighting, social schedules, and behavioral factors, leading to more complex outcomes. Therefore, conducting well-designed randomized controlled trials and longitudinal studies across different age groups and baseline refractive states will help generate evidence with greater clinical translational value for future light-related myopia prevention and control strategies.

## 7. Intervention: Translating Mechanisms into Clinical Practice

Based on positive evidence of light’s influence on myopia, several device-based intervention approaches have been explored or proposed to date.

Blue light therapy has been used to target the optic disc area for myopia treatment. Short-term blue light exposure slows axial elongation, even in eyes experiencing hyperopic defocus [103]. For a more precise therapeutical option, another study directed 480 nm blue light at the optic disc, corresponding to the physiological blind spot in humans, to ideally stimulate the majority of ipRGCs, thereby increasing melanopsin activation and increasing choroidal thickness, while minimizing potential effects on rods and cones [258]. In addition, stimulation of the optic disc with blue light results in significant reductions in refractive error and axial growth [174], although this study only consisted of three emmetropic and three myopia individuals. In contrast, another study suggests that short-term blue light stimulation of the optic disc does not produce the expected effects, whereas stimulation of the central retinal area temporarily enhances retinal ganglion cell activity [111]. Studies like these, which fail to demonstrate similar significant effects even when attempting to follow relatively similar protocols, indicate the need for independently replicated results when attempting to establish novel myopia prevention therapeutics. In addition, while studies stimulating the optic nerve head propose to function via ipRGC axons at the optic disc expressing melanopsin [258,259], there are still no studies providing direct evidence of functionally significant melanopsin levels at the human optic nerve head. In addition, one small study indicated that significant choroidal thickening following blue light stimulation of the blind spot only occurs in emmetropes, not myopes [173]. A recent clinical trial involving children (aged 6 to 12 years) using a smartphone mounted VR headset to stimulate the optic disc with blue light (NCT04967287) failed to demonstrate remarkable differences in axial length changes and spherical refraction after 12 months of treatment compared to control groups [260]. However, there were notable adherence complications and dropout rates in the intervention group, which may have influenced results. While blue light therapy may emerge as a potential treatment option, more consistent evidence and more rigorous, well-designed clinical trials are needed to determine ideal application methods and whether results are consistent among different refractive groups.

Recently, low-level red light therapy (LLRL) has emerged as a potential treatment option for myopia control. Preliminary studies have shown promising evidence, resulting in a clinical trial that is currently still ongoing at the time of publication (NCT05606237). In a long-term efficacy study of LLRL in Chinese children, axial elongation and spherical refraction progression were smallest in the group receiving 2 years of continuous LLRL treatment (*p* < 0.001 for both measures) [261]. However, there was a modest rebound effect observed after stopping treatment [261]. This finding is similar to that seen in the aforementioned study of Smith et al., in which the removal of long-wavelength-pass filters caused recovery from the induced hyperopia in rhesus monkeys. This raises questions as to the long-term efficacy of LLRL treatment. Differences in choroidal thickness measurements between short- and long-term treatments in different age groups have also been noted. In a study of LLRL treatments in children, choroidal thickening was seen at 1 month and persisted for up to 12 months [262]. Meanwhile, studies in young adults have shown that red light exposure leads to choroidal thinning in short-term measurements [103,263]. In another study, compared to younger participants, older children show slowed axial growth with LLRL treatment, but no statistically significant differences in choroidal thickness based on age [264], hinting that the mechanism modulating LLRL therapy’s influence on myopia may not involve direct changes to the choroid. These differences may be due to the age of the test subjects involved, but the conflicting findings still warrant further investigation.

In another study, twice daily, 30 min treatment with a head-mounted LLRL-emitting device resulted in a decrease in average axial length and average spherical refraction progression in children after three months of treatment [265]. Even just 3 min treatment sessions twice a day with LLRL in children has been shown to reduce axial elongation, reduce spherical refraction shift, and increase average choroidal thickness compared to controls [264]. In children at risk for myopia due to familial presence of high myopia, LLRL treatment resulted in reduced axial elongation, less myopic refractive drift, and a lower incidence of myopia after one year [266]. In children and adolescents with high myopia (≥−4.00 D in at least one eye), 12 months of LLRL treatment has shown notably stronger treatment efficacy [267]. Similarly, among children with myopia of −6.00 D or worse, 12 months of LLRL combined with single-vision glasses yielded an average reduction in axial length accompanied by increased choroidal thickness across all segments and thickening in some retinal regions [268]. Compared to orthokeratology, LLRL exhibits slightly superior efficacy in controlling axial elongation and choroidal thickness [264]. These studies provide a glimpse of how future LLRL treatments may be quite practical if only a short time span is necessary to produce results. However, the noted rebound effect indicates that there may be a necessary period of treatment to achieve a stable result over the long term, and variations in efficacy with age may indicate that there is an ideal treatment window.

When interpreting these findings, it is also important to note that many of the LLRL studies presented do not meet the IMI’s recommended standards for minimum study duration and washout period [269], which raises further concern about the long-term efficacy and potential rebound effect of treatment.

A small number of LLRL studies have reported adverse events. As mentioned in a previous section, since most myopia prevention therapies are being proposed for use in children, there are legitimate safety concerns raised about device usage. At lower concern levels, there have been reports of short periods of transient dizziness following treatment [270], as well as reversible vision loss due to flash blindness or glare with an afterimage [131,271]. Fundus photographs have also shown bilateral macular darkening with autofluorescence patches post-treatment, with OCT-identified bilateral ellipsoid zone disruption and discontinuity in the inter-foveal region [73]. As mentioned in a previous section, more severe complications such as binocular vision loss with foveal photoreceptor and RPE damage [73], as well as reduced foveal cone density [74], have been reported. Study protocols frequently implement inadequate methods for assessing adverse events [72], raising concerns about safety. Much more rigorous testing protocols and reporting standards are necessary to confirm both the safety profile and long-term results of LLRL. A currently ongoing clinical trial in the United States will hopefully better describe its potential clinical applications in due time.

While violet light exposure has been proposed as a myopia prevention strategy, there still is a general lack of human studies utilizing violet light-based treatment interventions. In a Japanese double-blind randomized clinical trial, violet light-transmitting eyeglasses were compared with conventional violet light-blocking eyeglasses. The results showed that during the two-year observation period, the violet light transmission group exhibited a smaller degree of axial elongation, consistent with previous reports of axial growth inhibition under intervention measures [61]. One trial demonstrated that in children aged 8 to 10 years old, wearing a violet light-emitting eyeglass frame for 3 h per day for 6 months reduced progression of myopia, slowed axial length growth, and thickened the choroid [59]. The irradiance of the emitted violet light was equal to the average daily irradiance levels in Tokyo and was considered generally safe [59]. However, statistically significant findings were not found in other age groups considered. In addition, since the eyeglass frames also incorporated a violet light sensor to measure outdoor light exposure, varying daily exposures could be taken into account when comparing the results [59]. This indicates that all participants were experiencing less than 1 h of UV light exposure per day [59]. As such, it remains to be seen whether adverse event outcomes may change with increasing daily outdoor time. These results are promising, even though the sample size was relatively small.

However, when considering light therapies, it is critical to consider not only the spectrum of light but also its intensity, pattern timing, and potential influence on the circadian system. The variation seen in clinical data may also be in part due to reduced spectral sensitivities to particular wavelengths that result in variation in findings for spectral-based studies [272]. There are also notable anatomical variances which may alter the intensity of light reaching the retina. For example, it is unknown whether differences in factors such as axial length or pupil size [273], which varies in myopes compared to emmetropes, may influence luminance levels at the retina. At the same time, it is also important to note that transmission levels of light change over time as the crystalline lens changes with age, and there have been noted differences in crystalline lens parameters in myopic eyes [274]. This may need to be taken into account when considering light-based therapies, especially spectrum-specific therapies. Age-related changes in the lens’s transmission properties may result in significantly reduced transmission of the target wavelengths, especially if the therapy involves shorter wavelengths [275].

Pharmacologic translation of light-linked molecular pathways is also being explored, including a recent proposal of topical dopamine as a potential myopia prevention treatment [276]. However, compared to light-based therapies, studies utilizing the proposed molecular pathways are much scarcer.

Behavioral interventions are also being explored through technological applications. For example, an “Eye-Use Monitoring” device has been developed to provide vibration alerts for poor eye usage habits in children, including low lighting conditions [277]. This can promote behavior changes which may slow the progression of myopia and reduce the incidence of myopia. However, maintaining these effects requires sustained intervention, and more studies are needed to confirm long-term benefits.

In summary, collectively, these device-based or pharmacologically based intervention approaches indicate that strategies utilizing light for myopia control are emerging and feasible, but careful consideration must be paid to individual differences and the prevention of adverse side effects.

## 8. Conclusions

Our understanding of how the eye interprets light has rapidly progressed over the past several decades. Far from only being used to form visual interpretations of our environment, light also influences a wide range of biological processes, including ocular development. The complexity of these interactions has led to a wide range of theories and interpretations of the mechanisms driving them, occasionally with conflicting explanations. As future research continues to explore photobiomodulation processes, the convoluted link between the epidemiological evidence supporting outdoor sunlight’s protective effects against myopia development and the molecular workings modulating ocular changes may be explained. At present, both the intensity of light and particular wavelengths of light may be critical in the emmetropization process. However, conflicting research findings indicate that there may be different spectral sensitivities between species, as well as varying distributions of retinal photoreceptors that can influence interpretation. While animal studies have been generally supportive, translating this evidence to the human condition remains challenging. A lack of robust evidence in human studies has made it difficult to establish any clinical guidelines regarding light exposure and myopia development. There is a great need for more RCTs to investigate what specific components of light, or combination of components, may contribute to anti-myopic development.

Similarly, while small-scale studies have indicated promising results for light-based therapies to prevent myopia progression, insufficient reporting methods and study protocols and a lack of large-scale study confirmations indicate that there is still a long way to go before these strategies can be implemented in clinical practice.

It has been suggested that the neurotransmitter dopamine serves as a “stop signal” for myopic development, and light exposure increases dopamine signaling within the retina. Decreased levels of dopamine and DOPAC within the retina have been associated with increased myopia, indicating that a dopamine-dependent pathway may be involved in emmetropization. However, varied research findings suggest that this is not the only molecular influence on myopia development, and much more research is needed to fully elucidate these pathways.

The nonvisual opsins OPN3, OPN4, and OPN5 may also be responsible for wavelength-specific modification of ocular growth processes, perhaps through pathways utilizing dopamine or EGR-1, but none of these studies can currently be translated to humans. Much is still unknown about these opsins, leaving an exciting new field of potential research opportunities.

The findings of photobiomodulation research may yield future benefits, both in and out of the clinic. Therapeutic strategies utilizing particular spectral wavelengths may help stem the tide of the myopia epidemic, and future research confirming safety and efficacy may lead to blue, violet, or red light-emitting devices being utilized either at home or in clinic on a regular basis. Future findings may also lead to updated regulations for indoor lighting or window transmission rates to permit specific wavelengths, as well as the implementation of school programs to promote more outdoor time to prevent myopia progression. However, in order for these practical and clinical recommendations to be established, much more rigorous research is needed. To improve comparisons between studies, clarification of both the illuminance as well as the spectral density of light sources utilized should be reported. In addition, particular attention to species-specific variances in ocular physiology and spectral sensitivity is needed when interpreting animal studies within this field. While an overwhelming number of studies have demonstrated a relationship between varying types of light and myopia development, efforts to elucidate the mechanisms of photobiomodulation are still lacking. Determining the molecular mechanisms driving this relationship is likely critical to explaining the variation in results seen between studies and developing the most effective treatment strategies regardless of age and refractive status.

In summary, it is clear that there is a connection between light exposure and myopia, and as the global prevalence of myopia continues to rise dramatically, it becomes even more pertinent to develop a comprehensive understanding of this link. While the past several decades of research have led to many exciting suggestions as to what the underlying mechanisms driving this relationship may be, there is still much remaining to be discovered. Photobiomodulation currently stands as an exciting frontier that may help shape future efforts to mitigate myopia and lead to strategies to prevent high myopia and reduce the global burden of visual impairment.

## Data Availability

No data was used in the writing of this review.

## Abbreviations

The following abbreviations are used in this manuscript:

LIM	Lens-induced myopia
COVID-19	Coronavirus disease 2019
FDM	Form-deprivation myopia
ERG	Electroretinography
OCT	Optical coherence tomography
IMI	International Myopia Institute
UV	Ultraviolet
LCA	Longitudinal chromatic aberration
NO	Nitric oxide
DOPAC	3,4-Dihydroxphenylacetic acid
MEL-SAP	Melanopsin–saporin
DAT	Dopamine transporter
VGAT	Vesicular GABA/glycine transporter
EGR-1	Early growth response-1
IOL	Intraocular lens
GPCRs	G-protein-coupled receptors
MPH	Methlyphenidate hydrochloride
atRA	All-trans retinoic acid
NOS	Nitric oxide synthase
cGMP	Cyclic GMP
LLRL	Low-level red light therapy
